# Novel insight into lepidopteran phylogenetics from the mitochondrial genome of the apple fruit moth of the family Argyresthiidae

**DOI:** 10.1186/s12864-023-09905-1

**Published:** 2024-01-02

**Authors:** Abdelhameed Elameen, Simo N. Maduna, Melissa H. Mageroy, André van Eerde, Geir Knudsen, Snorre B. Hagen, Hans Geir Eiken

**Affiliations:** 1 Division of Biotechnology and Plant Health, NIBIO, Norwegian Institute of Bioeconomy Research, Høghskoleveien 7, N-1431 Aas, Norway; 2 Division of Environment and Natural Resources, NIBIO, Norwegian Institute of Bioeconomy Research, Høghskoleveien 7, N-1431 Aas, Norway

**Keywords:** *Argyresthia conjugella*, Illumina HiSeq, Lepidoptera, Mitochondrial genome, Yponomeutoidea

## Abstract

**Background:**

The order Lepidoptera has an abundance of species, including both agriculturally beneficial and detrimental insects. Molecular data has been used to investigate the phylogenetic relationships of major subdivisions in Lepidoptera, which has enhanced our understanding of the evolutionary relationships at the family and superfamily levels. However, the phylogenetic placement of many superfamilies and/or families in this order is still unknown. In this study, we determine the systematic status of the family Argyresthiidae within Lepidoptera and explore its phylogenetic affinities and implications for the evolution of the order. We describe the first mitochondrial (mt) genome from a member of Argyresthiidae, the apple fruit moth *Argyresthia conjugella*. The insect is an important pest on apples in Fennoscandia, as it switches hosts when the main host fails to produce crops.

**Results:**

The mt genome of *A. conjugella* contains 16,044 bp and encodes all 37 genes commonly found in insect mt genomes, including 13 protein-coding genes (PCGs), two ribosomal RNAs, 22 transfer RNAs, and a large control region (1101 bp). The nucleotide composition was extremely AT-rich (82%). All detected PCGs (13) began with an ATN codon and terminated with a TAA stop codon, except the start codon in *cox1* is ATT. All 22 tRNAs had cloverleaf secondary structures, except *trnS1*, where one of the dihydrouridine (DHU) arms is missing, reflecting potential differences in gene expression. When compared to the mt genomes of 507 other Lepidoptera representing 18 superfamilies and 42 families, phylogenomic analyses found that *A. conjugella* had the closest relationship with the Plutellidae family (Yponomeutoidea-super family). We also detected a sister relationship between Yponomeutoidea and the superfamily Tineidae.

**Conclusions:**

Our results underline the potential importance of mt genomes in comparative genomic analyses of Lepidoptera species and provide valuable evolutionary insight across the tree of Lepidoptera species.

**Supplementary Information:**

The online version contains supplementary material available at 10.1186/s12864-023-09905-1.

## Background

Lepidoptera is the second largest insect order, with > 160,000 species [1]. This order includes both butterflies and moths, many of which are important model organisms in ecology and evolutionary biology [2]. In Lepidoptera, mitochondrial (mt) genomes are widely used to study population genetics, phylogeography, phylogenetics and molecular taxonomy [3–5]. In particular, the mitogenome represents an ideal tool for the analysis of phylogenetic relationships due to its simple structure, maternal inheritance, low recombination, and high conservation over the course of evolution [6, 7]. Mitogenomes may also provide information to identify novel genes that may serve as targets in future research [8]. The mitogenome size of Lepidoptera ranges from 15,000 bp to above 16,000 bp [6, 7], mostly due to the variable length of noncoding regions, particularly the control region [8]. Moreover, lepidopteran mt genomes have a conserved rich adenine and thymine (A + T) region and usually consist of 37 genes, encoding 13 conserved protein-coding genes (PCGs), 22 tRNAs, 2 rRNAs, and a noncoding control region [6, 7, 9]. Until recently, the bulk of Argyresthiidae phylogenetic analyses utilized a common set of 8–11 mitochondrial and nuclear genes [10] and a set of up to 27 protein-coding genes [11–16]. However, inadequate node support hindered research that attempted to unravel relationships among superfamilies, even with over 1500 genes [17–20]. The potential causes and consequences of the competing phylogenetic hypotheses were discovered to be compositional bias and other model violations [21].

Yponomeutoidea is a large superfamily of Lepidoptera with 11 families and 1800 species [16, 22]. Surprisingly, only seven mt genome species of this superfamily are available in databases [23]. For the Argyresthiidae family, which belongs to this superfamily and contains 157 species [16], no mt genomes exist. Thus, obtaining the mt genomes of this family may be warranted for further resolving patterns of genomic evolution and assessing phylogenetic relationships.

The apple fruit moth (*Argyresthia conjugella,* Zeller) has a wide circumpolar distribution [24, 25]. Its main host, rowan (*Sorbus aucuparia*), is a masting species with spatiotemporally synchronized crop output [26]. In heavy intermast years, the apple fruit moth can hatch to find no host material available and will therefore seek secondary hosts, causing serious damage to apple crops [27]. *A. conjugella* is known to have high genetic diversity and a wide distribution in Fennoscandia [28–30], but the lack of complete mitogenomes for *A. conjugella* and the family Argyresthiidae hampers further studies on systematics, population genetics, taxonomy and evolutionary biology.

Our primary aim was to characterize the first entire mitogenome of a species in the Argyresthiidae family using the apple fruit moth in Norway as our study organism. Second, we analysed genome structure, base composition, substitution, and evolutionary rates among superfamilies using previously published Lepidoptera mitogenomes to obtain a better understanding of the phylogeny of Lepidoptera. We hypothesized that our phylogenetic analysis would recover Argyresthiidae nested with Yponomeutoidea. Furthermore, we evaluated the phylogenetic hypothesis that Argyresthiidae shows a sister-group relationship with Lyonetiidae, i.e., the ‘AL’ clade (Argyresthiidae + Lyonetiidae) of Sohn et al. (2013) [16] obtained based on nuclear genes. Finally, we wanted to provide an up-to-date identification of source taxa of lepidopteran sequences lacking superfamily-, family-, and/or genus-level ID on GenBank using a phylogenetic systematics framework.

## Results and discussion

### Genome assembly

**Fig. 1:**
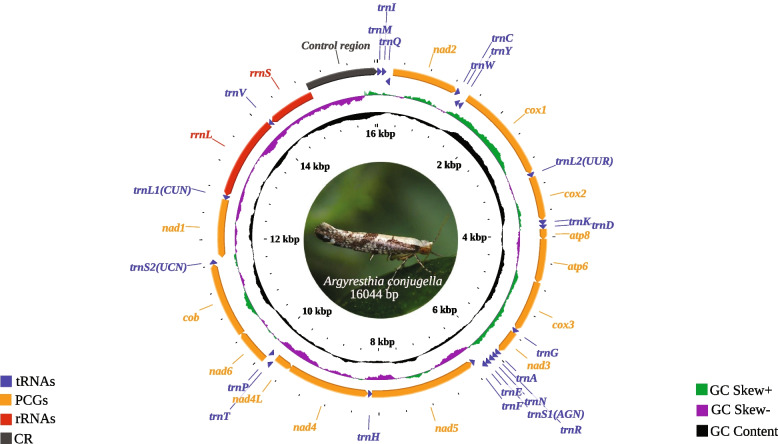
Circular map of the complete mitogenome of *Argyresthia conjugella* depicting gene order. Labelling of tRNA genes was conducted in accordance with IUPAC-IUB single-letter amino acid codes

Except for the variable control region (CR) and Norgal assemblies, we recovered the same gene order and content using both of our mitogenome assembly strategies. We discovered that Norgal failed to assemble mitogenome sequences when using the de novo assembly strategy, with no mitogenomic features (PCGs, tRNAs, and rRNAs) found for both assemblies resulting from using default (assembly size = 24,871 bp) and adjusted parameters (assembly size = 29,520 bp, -*m* 500). However, when run using the baited de novo assembly strategy, Norgal recovered the same gene order and content as SPAdes and Geneious Prime®, with the exception of the large ribosomal RNA gene (*rrnL*), which differed in size by 1 bp and sequence from that recovered by SPAdes and Geneious Prime® (pairwise *p*-distance = 0.01). We found that the variations in mitogenome sizes were associated with the properties of the control region (*CR*), which include variation in the copy number of tandemly repeated sequences and extensive length variation of a variable domain [31, 32]. When using SPAdes under the de novo assembly strategy, the nearly complete *CR* (1101 bp) was recovered. When the baited de novo assembly strategy was used, SPAdes recovered a partial *CR* of 380 bp in which the repetitive sequences could not be assembled. As a result, we present the complete mitogenome sequences of the apple fruit moth from the SPAdes de novo assembly, where the mitogenome is a 16,044 bp closed circular molecule (GenBank accession: ON496993; Fig. 1). Interestingly, the mitogenome size of the apple fruit moth was similar to available Yponomeutoid mitogenomes [33–35], which are relatively longer on average compared to other superfamilies of Lepidoptera (*n* = 4, 16092 ± 353 bp, Table 1).

**Table 1:** General information and nucleotide composition for a subset of 51 representative mitochondrial genomes of the order Lepidoptera and 5 Trichopteran outgroups used in this study

Superfamily	Family	Species	Accession number	Size (bp)	Whole genome composition						
					**T%**	**C%**	**A%**	**G%**	**A + T%**	**AT skew**	**GC skew**
Bombycoidea	Bombycidae	*Bombyx huttoni*	KP216766	15638	38.0	11.1	43.8	7.1	81.8	0.071	-0.220
	Sphingidae	*Ampelophaga rubiginosa*	KT153024	15, 82	40.1	11.0	41.4	7.5	81.5	0.016	-0.189
	Eupterotidae	*Ganisa cyanogrisea*	MF100140	15174	39.8	12.8	39.7	7.7	79.5	-0.001	-0.249
	Endromidae	*Prismosticta fenestrata*	MF100145	15772	39.9	11.2	41.2	7.6	81.1	0.016	-0.191
	Saturniidae	*Neoris haraldi*	MF664471	15343	40.3	12.4	39.4	7.9	79.7	-0.011	-0.222
	Brahmaeidae	*Brahmaea certhia*	MG018026	15429	40.2	11.6	40.7	7.5	80.9	0.006	-0.215
	Lasiocampidae	*Dendrolimus houi*	MK952760	15443	38.8	12.5	41.2	7.6	80.0	0.030	-0.244
Calliduloidea	Callidulidae	*Pterodecta felderi*	MT370823	15340	39.3	12.7	40.0	7.9	79.3	0.009	-0.233
Copromorphoidea	Carposinidae	*Carposina sasakii*	HQ840719	15611	39.5	10.7	42.0	7.8	81.5	0.031	-0.157
Cossoidea	Cossidae	*Zeuzera multistrigata*	MF491642	15260	39.2	13.1	39.7	7.9	78.9	0.006	-0.248
Drepanoidea	Drepanidae	*Oreta fuscopurpurea*	MG572766	15564	39.2	12.3	40.4	8.1	79.6	0.015	-0.206
	Epicopeiidae	*Epicopeia hainesii*	MK033610	15395	39.6	11.8	41.0	7.6	80.6	0.017	-0.216
Gelechioidea	Stathmopodidae	*Atrijuglans hetaohei*	KT581634	15379	42.1	11.1	39.3	7.6	81.4	-0.034	-0.187
	Scythrididae	*Scythris sinensis*	MH230111	15216	42.2	11.4	38.7	7.7	80.9	-0.043	-0.194
	Coleophoridae	*Coleophora therinella*	MH473596	15539	41.6	11.8	39.1	7.5	80.7	-0.031	-0.223
	Oecophoridae	*Opisina arenosella*	MK467611	15389	41.3	12.0	39.2	7.4	80.5	-0.026	-0.237
	Cosmopterigidae	*Meleonoma mirabilis*	MW366996	15268	40.0	14.8	37.1	8.2	77.1	-0.038	-0.287
	Lecithoceridae	*Issikiopteryx taipingensis*	ON323501	15234	41.5	12.4	38.4	7.7	79.9	-0.039	-0.234
	Gelechiidae	*Monochroa sp.*	ON323502	15299	40.9	12.5	39.0	7.7	79.9	-0.024	-0.238
Geometroidea	Uraniidae	*Acropteris iphiata*	MN093120	15346	37.9	11.5	43.1	7.5	81.0	0.064	-0.211
	Geometridae	*Amraica recursaria*	MZ713635	15609	39.8	11.0	41.6	7.6	81.4	0.022	-0.183
Hedyloidea	Hedylidae	*Macrosoma conifera*	NC_050856	15344	41.2	10.9	40.5	7.5	81.7	-0.009	-0.185
Hepialoidea	Hepialidae	*Endoclita signifer*	KT780172	15285	41.4	10.5	40.5	7.6	81.9	-0.011	-0.160
Hesperioidea	Hesperiidae	*Choaspes benjaminii*	JX101620	15272	40.7	11.7	40.1	7.5	80.8	-0.007	-0.219
Noctuoidea	Euteliidae	*Eutelia adulatricoides*	KJ185131	15360	40.7	11.3	40.2	7.8	80.9	-0.006	-0.183
	Nolidae	*Sinna extrema*	MG872330	15345	40.7	11.4	40.1	7.8	80.8	-0.007	-0.188
	Erebidae	*Asota caricae*	MZ779033	15409	40.3	12.2	40.0	7.5	80.3	-0.004	-0.239
	Erebidae	*Spilarctia casigneta*	MZ959068	15402	40.2	12.2	40.2	7.4	80.4	0.000	-0.245
	Noctuidae	*Acronicta rumicis*	NC_062117	15388	40.3	11.7	40.4	7.7	80.7	0.001	-0.206
	Notodontidae	*Syntypistis chambae*	NC_062123	15938	39.4	11.3	41.9	7.4	81.3	0.031	-0.209
Papilionoidea	Lycaenidae	*Cigaritis/Spindasis takanonis*	HQ184266	15349	41.0	10.7	41.3	6.9	82.3	0.004	-0.216
	Riodinidae	*Abisara fylloides*	HQ259069	15301	41.7	11.3	39.5	7.5	81.2	-0.027	-0.202
	Papilionidae	*Graphium chironides*	KP159289	15235	40.8	11.9	39.6	7.7	80.4	-0.015	-0.214
	Pieridae	*Colias erate*	KP715146	15184	41.4	11.2	39.9	7.5	81.3	-0.018	-0.198
	Nymphalidae	*Apatura laverna*	MF444860	15187	40.2	12.2	39.7	7.9	79.9	-0.006	-0.214
Pyraloidea	Thyrididae	*Pyrinioides aurea*	KT337662	15362	40.0	12.2	40.0	7.9	80.0	0.000	-0.214
	Pyralidae	*Orthaga olivacea*	MN078362	15174	41.2	12.8	37.8	8.2	79.0	-0.043	-0.219
	Crambidae	*Crambus perlellus*	NC_061606	15440	41.0	11.2	40.3	7.5	81.3	-0.009	-0.198
Sesioidea	Choreutidae	*Choreutis emplecta*	MG581932	14718	40.8	11.4	39.9	7.9	80.7	-0.011	-0.181
	Sesiidae	*Sesia siningensis*	MN708363	15454	38.6	12.5	41.0	7.9	79.6	0.030	-0.225
Tineoidea	Tineidae	*Amorophaga japonica*	MH823253	15027	39.4	10.9	42.3	7.5	81.7	0.035	-0.185
	Psychidae	*Dahlica ochrostigma*	MK890245	15429	39.1	11.1	42.8	7.0	81.9	0.045	-0.227
	Gracillariidae	*Gibbovalva kobusi*	MK956103	15717	39.5	11.6	41.0	7.9	80.5	0.019	-0.190
	Meessiidae	*Eudarcia gwangneungensis*	MN413148	15391	39.4	12.7	40.4	7.4	79.8	0.013	-0.264
Tortricoidea	Tortricidae	*Eudemis lucina*	MK820027	16,056	39.9	11.6	40.6	7.8	80.5	0.009	-0.196
Yponomeutoidea	Plutellidae	*Plutella xylostella*	JF911819	16079	40.5	10.9	40.9	7.7	81.4	0.005	-0.172
	Lyonetiidae	*Leucoptera malifoliella*	JN790955	15646	40.6	10.4	41.9	7.0	82.5	0.016	-0.195
	Praydidae	*Prays oleae*	KM874804	16499	40.1	10.7	41.7	7.5	81.8	0.020	-0.176
	Argyresthiidae	*Argyresthia conjugella*	ON496993*	16044	41.2	10.6	40.8	7.4	82.0	-0.005	-0.178
Zygaenoidea	Limacodidae	*Iragoides fasciata*	MK250437	15645	42.0	10.8	40.0	7.2	82.0	-0.024	-0.200
	Zygaenidae	*Amesia sanguiflua*	NC_046467	15203	39.8	12.4	40.0	7.8	79.8	0.003	-0.228
Hydropsychoidea	Hydropsychidae	*Cheumatopsyche brevilineata*	KX385010^a^	15302	38.6	14.5	39.2	7.7	77.8	0.008	-0.306
Limnephiloidea	Limnephilidae	*Limnephilus hyalinus*	NC_044710^a^	15168	38.4	13.9	39.5	8.1	77.9	0.014	-0.264
Phryganeoidea	Phryganeidae	*Phryganea cinerea*	NC_039714^a^	15039	39.1	14.0	39.2	7.7	78.3	0.001	-0.290
	Phryganopsychidae	*Phryganopsyche latipennis*	NC_043771^a^	15167	39.5	13.4	39.1	8.1	78.6	-0.005	-0.247
Stenopsychidae	Stenopsychinae	*Stenopsyche angustata*	NC_051529^a^	15371	35.0	15.5	41.6	7.8	76.6	0.086	-0.330

^*^This study

^a^Trichopteran outgroup

### Genome organization and base composition

**Fig. 2:**
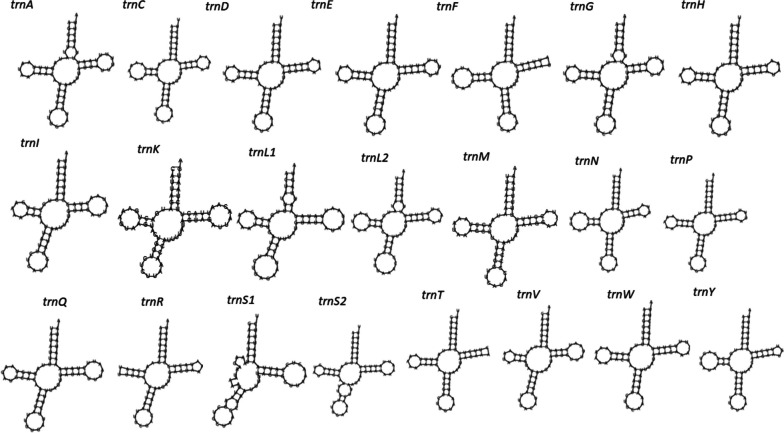
Predicted secondary structures of the 22 typical tRNA genes in *the A. conjugella* mitogenome

**Fig. 3:**
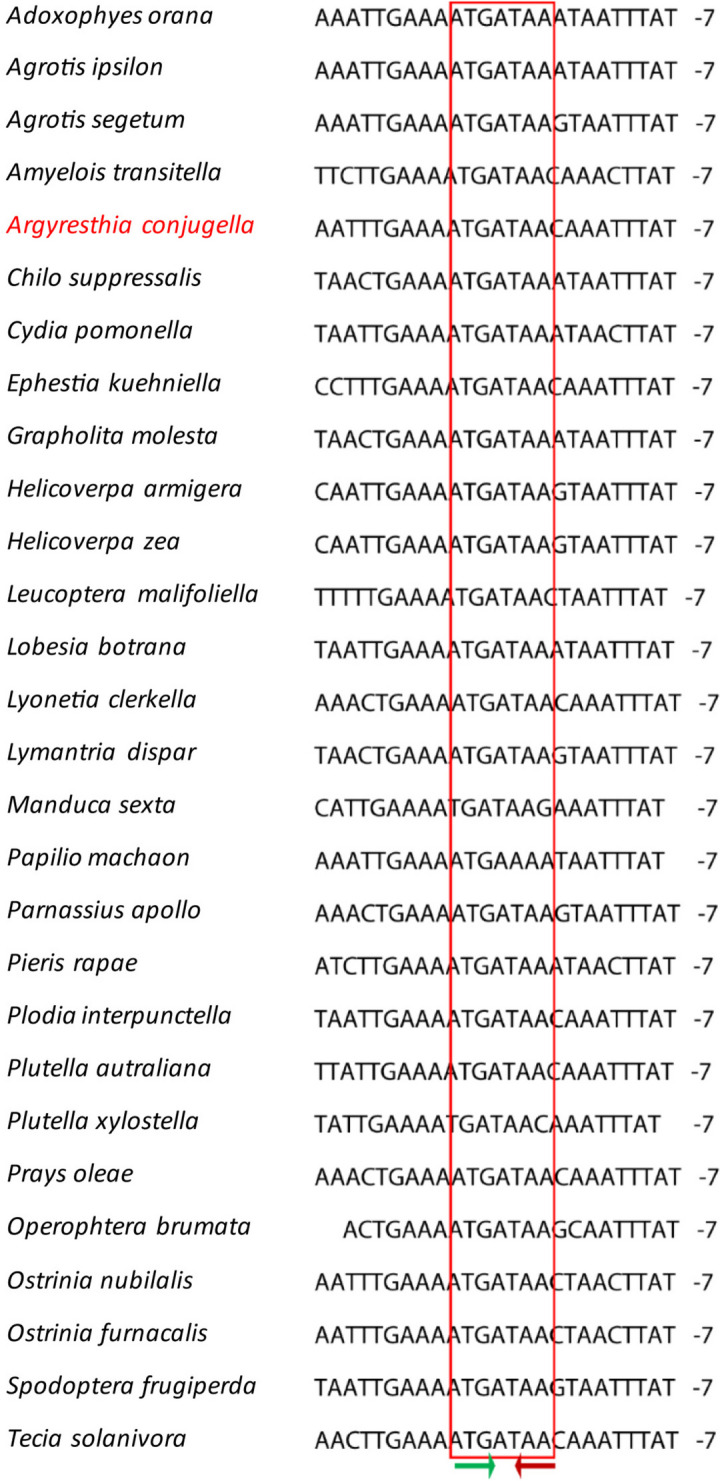
Alignment of *atp8* and *atp6* overlap of the selected lepidopteran species in the study, including *A. conjugella*. The green arrow shows the *apt6* start codon, and the red arrow shows the *atp8* stop codon

The gene content of the apple fruit moth mitogenome is similar to that of other Ditrysian insects studied previously, with 22 tRNA genes, 13 PCGs, 2 rRNAs and a noncoding control region. The low-strand codes for 9 PCGs (*cob*, *cox1, cox2, cox3, atp6, atp8, nad2, nad3* and *nad6*), 14 tRNAs (*trnM, trnI, trnW, trnL2, trnK, trnD, trnG, trnA, trnR, trnN, trnS1, trnE, trnT* and *trnS2*), 4 PCGs (*nad1*, *nad4*, *nad4L* and *nad5*), 8 tRNAs (*trnC*, *trnF*, *trnH*, *trnL1*, *trnP*, *trnQ*, *trnV*, *trnY*) and two mitochondrial rRNAs (*rrnL* and *rrnS*) (Fig. 1, Table 2). The lengths of the tRNA genes range from 64 to 75 bp (Table 2), which is well within the range of the corresponding tRNA genes of other lepidopterans: *Plutella xylostella* [34], *Parnassius apollo* [36], *Leucoma salicis* [7], *Ephestia kuehniella* [37] and *Speiredonia retorta* [6]. All 22 tRNAs had cloverleaf secondary structures, except *trnS1*, where one of the dihydrouridine (DHU) arms is missing (Fig. 2). The loss of the DHU arm in tRNAs has been detected in various Lepidoptera species [6, 38, 39]. DHU lacking arm was hypothesized to have evolved in response to recognition signals for seryl-tRNA synthetases, reflecting potential differences in gene expression [40, 41]. The location of *rrnL* is between *trnV* and *trnL1*, while *rrnS* is detected between the control region and *trnV*. These are the same gene positions found in *P. xylostella* [34]. The lengths of *rrnL* and *rrnS* in *A. conjugella* are 1371 bp and 783 bp, while the lengths of these genes are 1371 bp & 783 bp, 1344 bp & 840 bp and 1413 bp & 781 bp in *S. retorta*, *L. salicis* and *P. xylostella,* respectively [6, 7, 34]. The rRNA genes were A + T rich (82%), falling within the range detected in other Lepidoptera species, including *Agrotis segetum* [42], *Agrotis ip*s*ilon* [43], *Spodoptera frugiperda* [44], and *Papilio machaon* [45]. The rRNA AT and GC skewness values were found to be negative in most of the analyzed Lepidoptera mitogenomes in the study, including *A. conjugella*; however, in *Tecia solanivora* [46], *Spilarctia subcarnea* [47] and *S. retorta* [6], these values were positive. In *A. conjugella*, the *cox1* gene starts with ATT, which is different from the start codon in the superfamily Yponomeutoidea members *P. xylostella, Leucoptera malifoliella* and *Prays oleae*, where the gene start codon is CGA. The start codon of the *cox1* gene was found to be variable in other Lepidoptera species [48]. The size of this gene (1534) in *A. conjugella* is 3 bp larger than that in these three species (*P. xylostella, L. malifoliella* and *P. oleae*) in the same superfamily. The *cox*2 gene size (682 bp) is the same size as that of *L. malifoliella* but larger than that found in *P. xylostella* and *P. oleae* (679), while all these species have the size of the *cox3* gene (789 bp). The largest PCG found in *A. conjugella* mitogenomes is *nad5* (1732 bp), and the smallest one is *atp8* (162 bp). These results are widely reported in various insect mitogenomes [49, 50]. Overlap of the alginate sequences of *atp6* and *atp8* in *A. conjugella* (Fig. 3) showed the conserved nucleotide sequence ATG ATA A, which is detected in most lepidopteran species [34, 51].

**Table 2:** The organization and characteristics of the complete mitochondrial genome of *Argyresthia conjugella*

Gene	Strand	Position		Size		Codon		Anticodon	IGN
		**Start**	**End**	**size (bp)**	**Amino acid**	**Start**	**Stop**		
*trnM*	L	1	68	68				AUG	0
*trnI*	L	72	139	68				AUC	3
*trnQ*	H	135	205	71				CAA	-5
*nad2*	L	246	1259	1014	337	ATT	TAA		40
*trnW*	L	1261	1328	68				UGA	1
*trnC*	H	1321	1385	65				UGC	-8
*trnY*	H	1390	1455	66				UAC	4
*cox1*	L	1464	2997	1534	511	ATT	T		8
*trnL2*^*(UUR)*^	L	2994	3062	69				UUA	-4
*cox2*	L	3062	3743	682	227	ATG	TAA		-1
*trnK*	L	3743	3815	73				AAG	-1
*trnD*	L	3815	3881	67				GAC	-1
*atp8*	L	3881	4042	162	53	ATC	TAA		-1
*atp6*	L	4036	4712	677	225	ATG	TA		-7
*cox3*	L	4713	5501	789	262	ATG	TAA		0
*trnG*	L	5503	5570	68				GGA	1
*nad3*	L	5567	5923	357	118	ATT	TAA		-4
*trnA*	L	5935	6002	68				GCA	11
*trnR*	L	6000	6064	65				CGA	-3
*trnN*	L	6066	6131	66				AAC	1
*trnS1*^*(AGN)*^	L	6140	6207	68				AGC	8
*trnE*	L	6206	6274	69				GAA	-2
*trnF*	H	6311	6379	69				UUC	36
*nad5*	H	6379	8110	1732	577	ATT	TA		-1
*trnH*	H	8107	8174	68				CAC	-4
*nad4*	H	8173	9519	1347	448	ATG	TAA		-2
*nad4L*	H	9513	9800	288	95	ATG	TAA		-7
*trnT*	L	9803	9866	64				ACA	2
*trnP*	H	9866	9933	68				CCA	-1
*nad6*	L	9938	10468	531	176	ATT	TAA		4
*Cob*	L	10468	11622	1155	384	ATG	TAA		-1
*trnS2*^*(UCN)*^	L	11626	11692	67				UCA	3
*nad1*	H	11709	12656	948	315	ATG	TAA		16
*trnL1*^*(CUN)*^	H	12654	12728	75				CUA	-3
*rrnL*	H	12726	14096	1371					-3
*trnV*	H	14094	14161	68				GUA	-3
*rrnS*	H	14161	14943	783					-1
*Control region*		14944	16044	1101					

IGN values represent intergenic nucleotides and overlapping nucleotides ( −)

*H-strand* Heavy strand, *L-strand* Low strand

We found that the locations of the *trnM* gene follow the ditrysian type *trnM*-*trnI*-*trnQ* [52], which is different from non-ditrysian groups in Lepidoptera and from the ancestral order in which *trnM* is translocated: *trnI*-*trnQ*-*trnM* [52–54]. The control region of *A. conjugella* is large (1101 bp), which is a common feature detected in the superfamily Yponomeutoidea [35]. In comparison, the *CR* of the olive and diamondback moths were found to be ~ 1600 bp and ~ 1081 bp, respectively [34, 35]. We found that the *CR* is comprised of nonrepetitive sequences, including the motif ‘ATAGA’ followed by a 20 bp poly-T stretch, dinucleotide microsatellites (AT)_18_ and (AT)_53_, each flanked by ATTTA motifs, a (TAAA)_4_ adjacent to *trnM* instead of the 11 bp poly-A adjacent to tRNAs, and several imperfect repeat elements, indicating that the sequence in the present study may be partial. We found that the nucleotide composition of the *CR* was highly AT-rich, where the AT content was estimated at 94.3%, (A: 47.6%, T: 46.7%, G: 1.8%, C: 3.9%), where the AT skew was positive and the GC skews was negative, 0.010 and − 0.368, respectively. Overall, the nucleotide composition of the apple fruit moth mitogenome was also highly AT-rich, where the AT content was estimated at 82%, (A: 40.8%, T: 41.2%, G: 7.4%, C: 10.6%), and AT and GC skews were negative, − 0.005 and − 0.178, respectively (Table 1). These results are in agreement with results obtained in *P. xylostella* [34], *L. salicis* [7], *E. kuehniella* [37] and *S. retorta* [6].

**Fig. 4:**
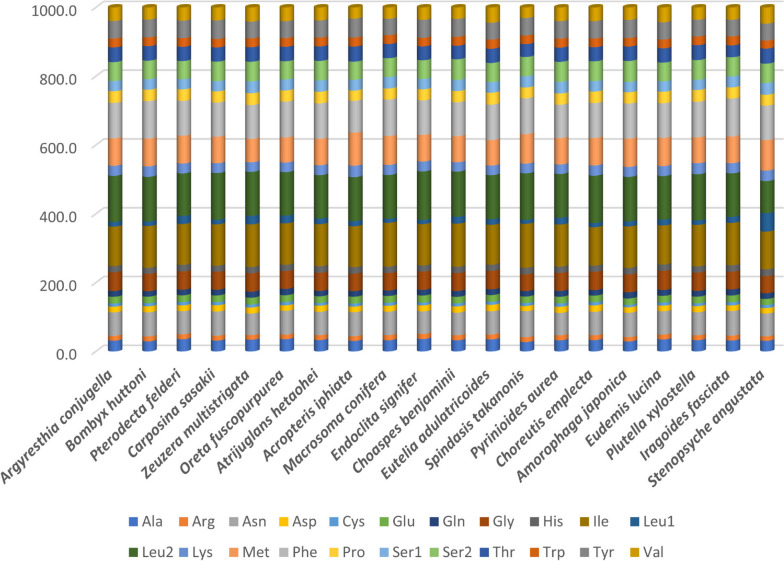
Comparison of codon usage of the 20 selected mitochondrial genomes of the Lepidoptera species in the study, including *A. conjugella*

**Fig. 5:**
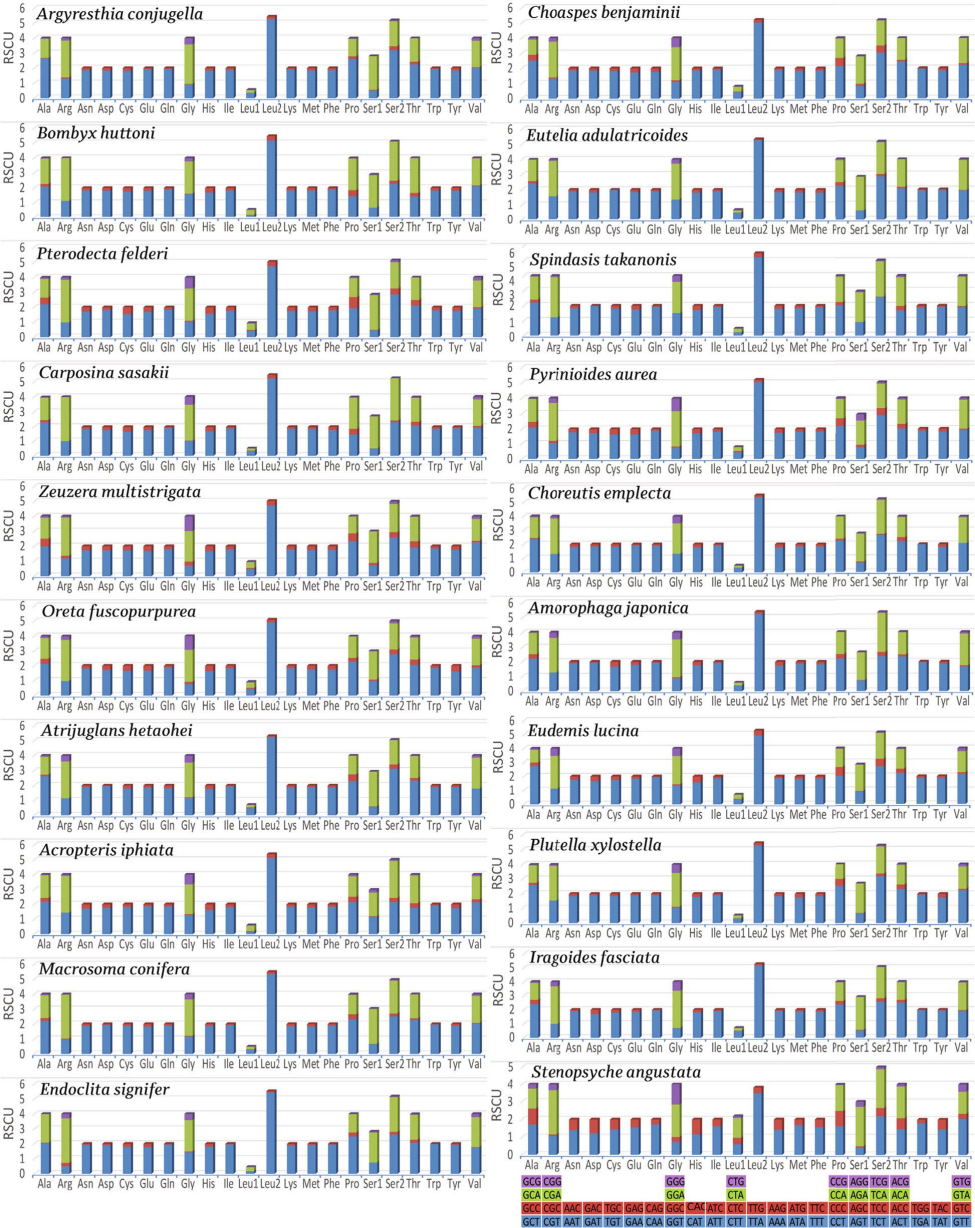
Relative synonymous codon usage (RSCU) of the 20 selected mitochondrial genomes of Lepidoptera in the study, including *A. conjugella*. Codon are plotted on the x-axis

**Fig. 6:**
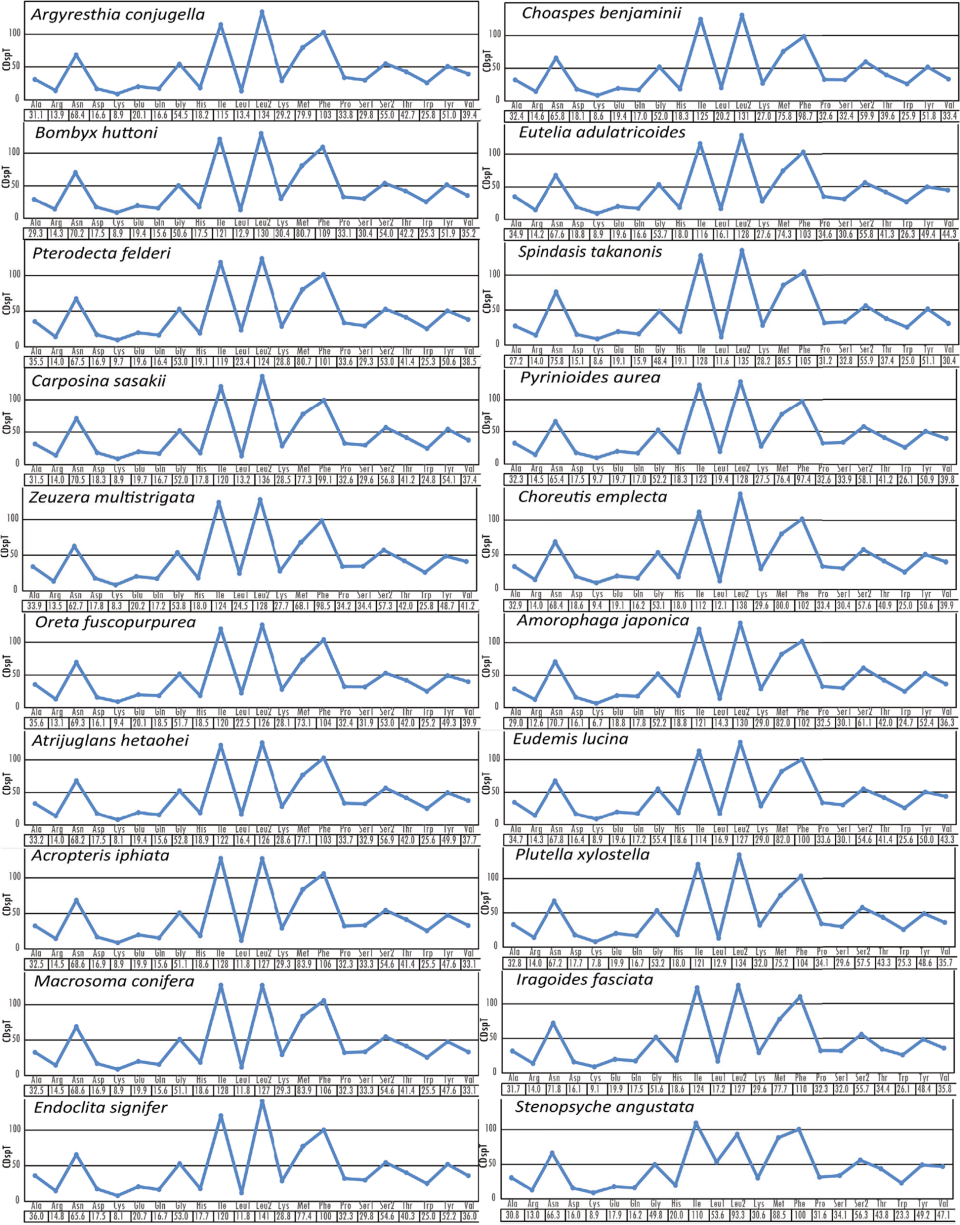
The distribution of codons among the selected lepidopteran species in the study. CDspT codons per thousand codons

The codon usage in *A. conjugella* was compared with twelve Lepidopteran species from different families (Fig. 4). The comparison showed that the pattern of codon usage in the PCGs of the *A. conjugella* mitogenome is very similar to the patterns in these Lepidopteran mitogenomes. Asn, Ile, Leu2, Met and Phe are the most commonly used codon families in all these species, while Cys codons are the rarest (Figs. 4 and 5). The relative synonymous codon usage (RSCU) was analysed for *A. conjugella* and compared with the same set of Lepidopteran insects (Fig. 6). CTG, CTC, AGG and ACG were completely absent in the *A. conjugella* mitogenome PCGs. Codons with high G and C contents are also rare or absent in the PCGs in other Lepidopteran mitogenomes. Moreover, TTA (Leu2), TCT (Ser2), CGT (Arg), GCT (Ala), and GGA (Gly) are the most frequently used codons and account for 36.41%. These five amino acids are also detected in other Lepidoptera species, such as *Manduca sexta* [55], *Helicoverpa armigera* [56], *P. xylostella* [34], *T. solanivora* [46], *P. machaon* [45], and *Ostrinia nubilalis* [57]. In particular, Leu2 was found to be the most frequently detected amino acid in all Lepidoptera species in the study, and this result is supported by results found in *L. salicis* [7] and *S. retorta* [6].

### Phylogenetics

**Fig. 7:**
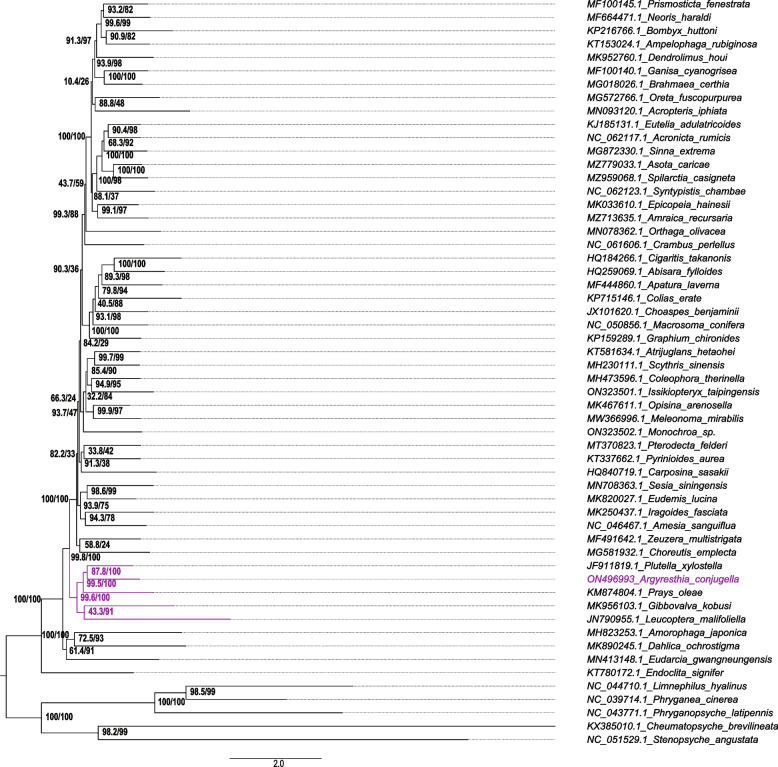
The phylogenetic tree included *A. conjugella*, constructed using the nearest neighbor interchange (NNI) approach to search for tree topology and for computing branch supports with 1000 replicates of the Shimodaira-Hasegawa approximate likelihood-ratio test *SH-aLRT* [68] and 1000 bootstrapped replicates of the ultrafast bootstrapping (*UFBoot2*) approach [69]. *Phryganea cinerea, Phryganopsyche latipennis, Cheumatopsyche brevilineata, Limnephilus hyalinus,* and *Stenopsyche angustata* were used as controls and outgroup species (Table 1)

To obtain an overview of *A. conjugella* and its relationships with other Lepidoptera species, our study investigated 18 superfamilies representing 42 families and 507 Lepidoptera species (Tables S1, S2 and Figure S2). This is the first phylogenetic study (using the mt genome) of *A. conjugella* in the Argyresthiidae family, which belongs to the Yponomeutoidea superfamily. Various studies tried to resolve phylogenetic tree of Lepidoptera using mitochondrial genomes, nucleotide alignments, amino acid alignments and transcriptomes and target enrichment approaches [6, 7, 9, 17–21, 58]. However, inadequate node support hindered research that attempted to unravel relationships among superfamilies [17–21]. The challenges are not the lack of data but, how to the data analyze, the quality of data and the number of taxon investigated [18, 21]. We constructed a phylogenetic tree using 507 Lepidopetera species (Fig. S2), and the subset data using 51 species (Fig. 7) to understand the position of *A. conjugella* in Lepidoptera phylogenetic tree. Using the ML approach, analyses of the three datasets (specified in the materials & methods section) resulted in the generation of three topologies. Generally, our study agrees with the most updated study Rota et al. (2022) [21], that detected nine main clades superfamilies in a butterfly and moth phylogeny using 331 genes for 200 taxa. Additionally, our phylogenetic analysis supports the previous morphological characterization of the Yponomeutoidea superfamily [16, 59, 60]. The 507 Lepidoptera species showed that some families clustered together, such as Papilionidae & Pieridae, Pyralidae & Tortricidae, Geometridae & Sphingidae, Erebidae & Noctuidae and Gelechiidae & Sphingidae, while other families as Tortricidae and Crambidae clustered alone and separately. Yponomeutoidea was recovered as a well-supported monophyly group and as one of the earliest lepidopteran groups after Tineoidea and the basal Hepialoidea (Fig. 7, Figures S1 and S2). However, the paraphyletic Tineoidea to some extent led to the phylogenetic instability of the monophyly of Yponomeutoidea in cases of Datasets 1 and 2 (Fig. 7, Figure S1), which was fully resolved with dense taxon sampling (Figure S2). Wang et al. (2018) [61], Bao et al. (2019) [38], Jeong et al. (2022) [23] and Zhang et al. (2020) [62], all found similar results for Yponomeutodiea and Tineoidea superfamilies. Furthermore, Boa et al. (2019) [38] and Jeong et al. (2022) [23] also found that Yponomeutoidea, Tineoidea and Gracillarioidea in Ditrysia have strong phylogenetic relationships. We also detected strong relationships between Yponomeutoidea, Zygaenidae and Tortricoidea, findings that are in line with results found by Liu et al. (2016) [48], Zhang et al. (2020) [62], Wang et al. (2018) [61], and Kim et al. (2014) [63]. Only a weak phylogenetic relationship was observed between the superfamilies Yponomeutoidea and Bombycoidea, results that are supported by Liu et al. (2016) [64] and Liu et al. (2017) [65]. Nonetheless, we consistently recovered Argyresthiidae embedded in Yponomeutoidea with a sister-group relationship to Plutellidae (Dataset 1: *SH-aLRT* = 92, *UFBoot2* = 100; Dataset 2: *SH-aLRT* = 88, *UFBoot2* = 100; Dataset 3: *SH-aLRT* = 87, *UFBoot2* = 99). Our phylogenetic tree hypothesis rejects the provisional ‘AL’ clade (Argyresthiidae + Lyonetiidae) recovered with nuclear gene datasets by Sohn et al. (2013) [16]. We found that Lyonetiidae was unstable, possibly due to its relatively long branch length. We recovered Lyonetiidae as basal to the Yponomeutoidea clade (Figure S1, Dataset 1: *SH-aLRT* = 99, *UFBoot2* = 100) or as a sister-group to Praydidae with Yponomeutoidea (Figure S2, Dataset 3: *SH-aLRT* = 84, *UFBoot2* = 100), and as sister-group to Gracillariidae of the order Tineoidea, although with weak support (Fig. 7, Dataset 2: *SH-aLRT* = 43, *UFBoot2* = 91). With increased taxon sampling, our phylogenetic tree hypotheses strongly supported the basal placement of Lyonetiidae within the Yponomeutoidea clade (Fig. 7, Figure S2, Dataset 2: *SH-aLRT* = 98, *UFBoot2* = 99). Moreover, we consistently recovered the previously described pairing of Yponomeutoidea and Gracillariidae as internested subclades [16, 22]. At a higher level, our phylogenetic tree hypothesis recovers some fundamental and uncontroversial lepidopteran clades that agree with the majority of mitogenomic phylogenies as well as those that included both mitochondrial and/or nuclear markers. The analyses found that *A. conjugella* had the closest relationship with *P. xylostella, L. malifoliella* and *P. oleae*, which belong to the Plutellidae, Lyonetiidae and Praydidae families, respectively (Fig. 7, Figure S2). Wei et al. (2013) [34], Sohn et al. (2013) [16], Liu et al. (2016) [48], Yang et al. (2020) [66], Jeong et al. (2021) [67] and Jeong et al. (2022) [23] all found that *P. xylostella, L. malifoliella and P. oleae* are closely related.

In our study, Tineodiea superfamily was represented by four species (*Amorophaga japonica, Dahlica ochrostigma, Gibbovalva kobusi* and *Eudarcia gwangneungensis*) with relatively high nodal support (Fig. 7, Figure S2). This superfamily is known to have high genetic diversity and has three different lineages [21]. The crosstalk of the complexity and the relationships among Tineidae group and the disagreements within the superfamily Gelechioidea*,* Carposinoidea*,* and Pterophoroidea remain unresolved issues*.* Both this study and that of Rota et al. (2022) [21], detected a sister relationship between Yponomeutoidea and the superfamily Tineidae, and the sub-clades Gelechioidea, Tortricoidea, Zygaenoidea are clustered together in the same clade. Rota et al. (2022) [21], found Gelechioidea clustered at different positions, when different analyses were performed with different datasets, these may be explained by high amount of compositional heterogeneity, or the limited materials used in the study (five species). While our study showed, the 20 species from Gelechioidea superfamily were clearly clustered together using both datasets and data two analyses (EME and NJ), but surprisingly, one single species (*Periacma orthiodes*) belonging to the superfamily Noctuoidea was clustered together with this family. This might be misidentification of the taxon of the mt genome found in the genebank. Our study showed, Gelechioidea grouped together with Pyralidae, these results are in agreement with the results of [17]. Pyaloidea was also sister to Carposinoidea; and Calliduloidea, Pterophoroidea, Gelechioidea and Thyridioidea are recovered in the same part of the tree, but with Thyridoidea sister to Macroheterocera [21]. Previously, Pterophoroidea was reported as a sister group with a monophyletic Papilionoidea, included Hedyloidea and Hesperioidea. In the same study, Choreutoidea and Immoidea were recovered as sister to Tortricoidea [21]. However, when 50 genes were removed, Choreutoidea were recovered as sister to Urodoidea and Pterophoroidea [21]. One phylogenetic study reported Pterophoroidea within the clade Obtectomera, [19] but a more recent study showed results contrary to these findings [21]. The position of Pterophoroidea is highly dependent on the dataset. This superfamily is recovered in the same clade with Urodoidea regardless of the alignment analysed, whereas it’s recovered in the clade with Gelechioidea, Calliduloidea and Thyridoidea is dependent on which datasets are analysed [21]. Pterophoroidea can also be recovered as sister to Papilionoidea and Noctuoidea, when different datasets were used [70]. It should also be noted that, using software with systematic errors and alignment issues can persist with regard to detecting homologies due to use of designed to assess the alignment quality using a threshold of alignment scores, [71].

Comprehensive analyses of insect mitogenomes provide important phylogenetic information to identify potentially novel genes that may serve as valuable targets in future research efforts. Further investigations of the whole genome of *A. conjugella* along with other genomes of Lepidoptera species will facilitate the understanding of the taxonomy and evolutionary process acting on the Ditrysia natural group.

## Materials and methods

### Specimen collection and DNA extraction

During August 2016, we collected a single female apple fruit moth larva from an infested rowan berry in the field in Skiftenes (N 6471746 and E 472502) in southern Norway. To confirm species identification of the larva, we employed both morphological [24, 65] and molecular methods [28] using microscopy and STR markers, respectively. We placed the apple fruit moth larva on rolls of corrugated cardboard until it entered pupal diapause, and then we stored it at -80 °C until DNA was extracted. DNA was extracted from the apple fruit moth pupal tissue using the DNeasy Blood and Tissue Kit (Qiagen, Tokyo) following a modified version of the manufacturer’s instructions [28].

### Mitogenome sequencing and assembly

We outsourced the whole genome sequencing of the apple fruit moth to the Norwegian Sequencing Centre (Oslo, Norway), where the whole genome library was prepared (insert size = 350 bp) and sequenced on one lane of the Illumina HiSeq 4000 platform (Illumina, USA) with paired end (PE) sequencing (2 × 150 bp). A total of 820,368,162 raw reads (of which 820,365,390 were paired in sequencing, *i*.*e*., 410,182,695 PE read clusters) were generated. We evaluated the quality of the Illumina sequencing run using MultiQC v.2.31 [72]. Then, we used AdapterRemoval v.2.1.3 to search for and remove adapter sequences and to trim low-quality bases from the 3' end of reads following adapter removal [73, 74]. After quality control (QC), we used the cleaned PE reads for mitogenome assembly by means of two assembly strategies: (i) de novo and (ii) ‘baited’ de novo.

For de novo sequence assembly (i), we employed the programs Norgal v.1.0 [75] and SPAdes v.3.15.3 [76]. The Norgal assembler was executed with (1) default parameters and (2) a *k*-mer range of 21–255 with an interval of 28 and a contig length threshold of 500 bp. We executed SPAdes for all *k*-mer sizes from 21 to 127 (-*k* 21, 33, 55, 77, 99, 127), with *–careful* option to minimize number of mismatches in the final contigs. For the baited de novo assembly strategy (ii), we first constructed the FM-index for the mitogenome reference sequence of the olive moth *P. oleae* (Bernard, 1788; Lepidoptera: Yponomeutoidea: Praydidae) (NCBI accession number NC_025948.1; [35] using the index command of the BWA v.0.7.17 aligner [77]. Additionally, we also used the mitogenome reference sequence of the diamondback moth *P. xylostella* [34] (Linnaeus, 1758; Lepidoptera: Yponomeutoidea: Plutellidae) (NCBI accession number JF911819.1). We selected the mitogenomes of the olive and diamondback moths as references due to their completeness and taxonomic and phylogenetic placement in Yponomeutoidea and the reliability of the PCR-based amplification method used to sequence these mitogenomes, 14 segments of 1.2–2.4 kb and nine segments of variable size, respectively. Second, we aligned the cleaned PE reads of the apple fruit moth separately to the indexed reference genome of the ‘model’ moths using the BWA-MEM algorithm of BWA, excluding reads with a minimum quality score of < 30, and then used the SAMtools v.1.9 suite [78] to convert the SAM to BAM alignment file. Third, we sorted and indexed the BAM alignment file using the *sort* and *index* commands, respectively, from SAMtools. Fourth, we obtained the QC statistics for the sorted and indexed alignment using BAMQC as implemented in Qualimap v.2.2.20 [79]. Fifth, we extracted reads that mapped properly as pairs using SAMtools. Finally, we used the mitochondrial filtered reads for de novo mitochondrial genome assembly using Norgal, SPAdes and Geneious Prime® v.2022.1.1 (Biomatters Ltd., Auckland, New Zealand; [80].

### Mitogenome annotation and visualization

We conducted a preliminary annotation of the mitogenome assembly referring to the results of the MITOS2 webserver (http://mitos2.bioinf.uni-leipzig.de/index.py; [81] Donath et al. 2019) by assessing the location of protein coding (PCGs), transfer RNA (tRNAs), and ribosomal RNA genes (rRNAs). Then, we confirmed gene boundaries for PCGs and rRNAs manually using BLASTn, SMART BLAST, BLASTp, and ORF Finder as implemented at the National Center for Biotechnology Information (NCBI) database [82]. Subsequently, we also validated that coding sequences were translated in the correct reading frame and confirmed the initiation and termination codons in Geneious Prime® using the published mitochondrial genome sequences of other moths as references, including the olive moth. We then used the program ARWEN [83] to detect the tRNA genes of the apple fruit moth and finally predicted the secondary structures of tRNAs using MITFI [84] as implemented in MITOS2 and tRNAscan-SE v.2.0 [85]. We also annotated the control (A + T-rich) region (*CR*) of the apple fruit moth by screening for structural elements characteristic of the region, which include (i) tandem repeats, identified using Tandem Repeats Finder v.4.10 [86] using default settings, and (ii) the motif ‘ATAGA’ and poly-T stretch. We produced the annotated circular map of the complete mitochondrial genome of the apple fruit moth using the beta version of the CGview server (http://cgview.cahttp://cgview.ca; [87]. The secondary structure of tRNAs was predicted using tRNAscan-SE-2.0 [88].

### Comparative mitogenomics of Lepidoptera

We conducted a systematic and comprehensive search for complete mitochondrial genomes of Lepidopteran species published in the NCBI nucleotide database using the following keywords: (“lepidoptera”[Organism] OR “lepidoptera”[All Fields]) AND “complete mitochondrial genome”[All Fields] AND mitochondrion[filter] (10 May 2022: 842 hits). We downloaded and processed the full GenBank files in Geneious Prime® to (i) obtain taxonomy metadata, (ii) remove least recently modified duplicates, (iii) remove nonlepidopteran species, and (iv) remove mitogenomes with > 90% missing annotations (retained 507 species). To ensure that the taxonomic status of all species was the latest, we verified all the species names against 60 taxonomic databases, including the Catalogue of Life and the Integrated Taxonomic Information System (ITIS), using the R package *taxize* v.9.94.91 [89]. Then, we corrected any misspellings and used the *classification* function implemented in *taxize* to retrieve the taxonomic ranks of individual species. We included seven species of Trichoptera, representing five families and four superfamilies, to serve as outgroups.

We compared the assembled *A. conjugella* mitochondrial genome with the mitochondrial genomes of 507 other Lepidoptera obtained from GenBank, representing 18 superfamilies and 42 families (Supplementary Table S2). We included only one representative per valid species (longest mitogenome sequence) when more than one known sequence was available in GenBank. We calculated the overall composition of individual mitogenomes based on the proportion of A + T out of the total (%AT content) using MEGA v.11.0.11 [90]. To measure the base composition skewness of nucleotide sequences, we used the formulae of Perna and Kocher (1995) [91]: AT-skew = [A-T]/[A + T] and GC-skew = [G-C]/[G + C].

### Sequence alignment and phylogenetic reconstruction

We produced codon-aware multiple sequence alignments for each of the 13 PCGs using MACSE v.2.01 [92]. We inspected and manually trimmed each set of alignments using MEGA, and any remaining ambiguously aligned sites were then further trimmed using BMGE v.1.12.1, with a sliding window size of 3 and maximum entropy of 0.5 [93]. We aligned rRNA genes using the online version of MAFFT v.7.299 [94, 95] and removed ambiguously aligned sites using BMGE. Before phylogenetic analysis, we produced two concatenated mitogenomic datasets from (i) the aligned individual PCG datasets (Dataset 1: *13PCGs_NT* dataset) and (ii) the 13 PCGs plus the large and small mitochondrial ribosomal RNA (rRNA) genes (*rrnL* and *rrnS*) (Dataset 2: *13PCGs_rRNAs_NT* dataset) with the R package *concatipede* v1.0.1 [96]. We derived the third mitogenomic dataset by translating the *13PCGs_NT* dataset in MEGA (Dataset 3: *13PCGs_AA* dataset). Furthermore, we used DAMBE v.7.2.141 [97] to conduct two-tailed tests of substitution saturation [98] for each codon position of the 13 PCGs, taking into account the proportion of invariant sites as recommended by Xia and Lemey (2009) [99]. According to the observed index of substitution saturation (*I*_*SS*_), all codon positions showed little saturation (*I*_*SS*_ < *I*_*SS*_*cSym* (assuming a symmetrical topology) and *I*_*SS*_ < *I*_*SS*_*cAsym* (assuming an asymmetrical topology); see Supplementary Table S2). Likewise, visual inspection of nucleotide saturation for each codon position of the 13 PCGs with DAMBE by plotting transitions and transversions against Kimura two-parameter [100] distances showed little saturation in all codon positions. Therefore, none of the codon positions were excluded, and the 13 PCG nucleotide (Dataset 1) and protein (Dataset 3) datasets were initially gene-by-codon partitioned (39 partitions) and gene partitioned (13 partitions), respectively. For Dataset 2, we designated two partitions for the rRNA genes (*rrnS* and *rrnL*, treated each as a single partition) and 39 partitions covering the three codon positions in each of the 13 protein-coding genes.

We used ModelFinder [101] to select the best-fitting partitioning scheme and models of evolution using the corrected Akaike Information Criterion (*AICc*) and the edge-linked proportional partition model [102] as implemented in IQ-Tree v. 2.2.0.3 [103]. We applied the new model selection procedure (*-m MF* + *MERGE*), which additionally implements the FreeRate heterogeneity model inferring the site rates directly from the data instead of being drawn from a gamma distribution (*-cmax* 20; [104]. To reduce the computational burden, the top 30% partition merging schemes were inspected using the relaxed clustering algorithm (*-rcluster* 30), as described in [105].

We reconstructed phylogenies based on the maximum likelihood (ML) criterion in IQ-Tree, where we used the substitution models indicated by ModelFinder (Table 3). We used the nearest neighbor interchange (NNI) approach to search for tree topology and for computing branch supports with 1000 replicates of the Shimodaira-Hasegawa approximate likelihood-ratio test *SH-aLRT* [68] and 1000 bootstrapped replicates of the ultrafast bootstrapping (*UFBoot2*) approach [69]. We abided by the advice that clades with *UFBoot2* ≥ 95 and *SH-aLRT* ≥ 80 can be regarded as being well supported [106].

**Table 3:** The substitution model MODELFINDER [101] was used to reconstruct phylogenies based on the maximum-likelihood (ML) criterion in IQ-TREE

Full data (516 taxa)		Subset data (56 taxa)	
**Partition**	**Model**	Partition	Model
*(a) Dataset 1 (13PCGs_NT)*			
*nad2*_pos1	GTR + F + R9	*nad2*_pos1	GTR + F + R5
*nad2*_pos2	GTR + F + R6	*nad2*_pos2	GTR + F + I + I + R4
*nad2*_pos3	GTR + F + ASC + R8	*nad2*_pos3	K3Pu + F + R6
*cox1*_pos1	GTR + F + I + G4	*cox1*_pos1	GTR + F + I + I + R4
*cox1*_pos2_*atp6*_pos2	GTR + F + I + I + R8	*cox1*_pos2	TVM + F + I + I + R3
*cox1*_pos3_*nad3*_pos3	GTR + F + I + I + R20	*cox1*_pos3_*cox2*_pos3_*cox3*_pos3_*nad6*_pos3_*cob*_pos3	TVM + F + R8
*cox2*_pos1	GTR + F + I + G4	*cox2*_pos1	GTR + F + I + I + R5
*cox2*_pos2	GTR + F + I + G4	*cox2*_pos2_*atp6*_pos2	GTR + F + I + I + R3
*cox2*_pos3_*atp6*_pos3	GTR + F + R20	*atp8*_pos1_*nad6*_pos1	TIM2 + F + I + G4
*atp8*_pos1	GTR + F + I + G4	*atp8*_pos2_*nad6*_pos2	GTR + F + R5
*atp8*_pos2	GTR + F + I + G4	*atp8*_pos3	K3Pu + F + R3
*atp8*_pos3	GTR + F + I + G4	*atp6*_pos1_*cob*_pos1	GTR + F + I + I + R5
*atp6*_pos1_*cox3*_pos1	GTR + F + R10	*atp6*_pos3_*nad3*_pos3	TPM2u + F + I + I + R6
*cox3*_pos2	GTR + F + I + G4	*cox3*_pos1	GTR + F + I + G4
*cox3*_pos3	GTR + F + ASC + G4	*cox3*_pos2_*nad3*_pos2_*cob*_pos2	GTR + F + I + I + R4
*nad3*_pos1	GTR + F + I + G4	*nad3*_pos1	GTR + F + I + I + R3
*nad3*_pos2	GTR + F + I + G4	*nad5*_pos1_*nad1*_pos1	GTR + F + I + I + R5
*nad5*_pos1_*nad1*_pos1	GTR + F + R9	*nad5*_pos2_*nad1*_pos2	GTR + F + I + I + R4
*nad5*_pos2_*nad1*_pos2	GTR + F + I + I + R7	*nad5*_pos3	GTR + F + I + I + R6
*nad5*_pos3	GTR + F + I + G4	*nad4*_pos1_*nad4L*_pos1	GTR + F + R4
*nad4*_pos1	GTR + F + I + G4	*nad4*_pos2	GTR + F + R5
*nad4*_pos2	GTR + F + I + G4	*nad4*_pos3	TVM + F + I + I + R6
*nad4*_pos3	GTR + F + ASC + G4	*nad4L*_pos2	TIM + F + I + I + R3
*nad4L*_pos1	GTR + F + I + G4	*nad4L*_pos3	TVM + F + R4
*nad4L*_pos2	GTR + F + I + G4	*nad1*_pos3	TIM + F + R5
*nad4L*_pos3	GTR + F + ASC + G4		
*nad6*_pos1	GTR + F + I + G4		
*nad6*_pos2	GTR + F + I + G4		
*nad6*_pos3	GTR + F + I + G4		
*cob*_pos1	GTR + F + I + G4		
*cob*_pos2	GTR + F + I + G4		
*cob*_pos3	GTR + F + ASC + G4		
*nad1*_pos3	GTR + F + ASC + G4		
*(b) Dataset 2 (13PCGs_rRNAs_NT)*			
*nad2*_pos1	GTR + F + R9	*nad2*_pos1	GTR + F + R5
*nad2*_pos2	GTR + F + I + I + R7	*nad2*_pos2	GTR + F + I + I + R4
*nad2*_pos3	GTR + F + ASC + R9	*nad2*_pos3	GTR + F + R5
*cox1*_pos1	GTR + F + I + I + R7	*cox1*_pos1	GTR + F + I + I + R5
*cox1*_pos2_atp6_pos2	GTR + F + R7	*cox1*_pos2	TVM + F + I + I + R3
*cox1*_pos3_cox2_pos3	GTR + F + I + I + R20	*cox1*_pos3_*cox2*_pos3_*nad6*_pos3_*cob*_pos3	TVM + F + R6
*cox2*_pos1	GTR + F + I + I + R6	*cox2*_pos1	GTR + F + I + I + R4
*cox2*_pos2	GTR + F + I + I + R6	*cox2*_pos2_*atp6*_pos2	GTR + F + I + I + R3
*atp8*_pos1	TIM3 + F + I + I + R5	*atp8*_pos1_*nad6*_pos1	TIM2 + F + I + G4
*atp8*_pos2	GTR + F + I + I + R5	*atp8*_pos2_*nad6*_pos2	GTR + F + R5
*atp8*_pos3	K3Pu + F + R6	*atp8*_pos3	K3Pu + F + R3
*atp6*_pos1_*cox3*_pos1	GTR + F + R10	*atp6*_pos1_*cob*_pos1	GTR + F + I + I + R4
*atp6*_pos3	TIM2 + F + R15	*atp6*_pos3_*nad3*_pos3	TPM2u + F + I + I + R5
*cox3*_pos2	TVM + F + I + I + R7	*cox3*_pos1	GTR + F + I + G4
*cox3*_pos3	GTR + F + ASC + R9	*cox3*_pos2_*nad3*_pos2_*cob*_pos2	GTR + F + R4
*nad3*_pos1	GTR + F + I + I + R6	*cox3*_pos3	K3Pu + F + R6
*nad3*_pos2	TVM + F + I + I + R6	*nad3*_pos1	GTR + F + I + I + R3
*nad3*_pos3	GTR + F + I + I + R10	*nad5*_pos1_*nad1*_pos1	GTR + F + R5
*nad5*_pos1	TVM + F + I + I + R10	*nad5*_pos2_*nad4*_pos2	GTR + F + R5
*nad5*_pos2_*nad1*_pos2	GTR + F + I + I + R7	*nad5*_pos3	K3Pu + F + R5
*nad5*_pos3_*nad4L*_pos3	TIM + F + I + I + R20	*nad4*_pos1_*nad4L*_pos1	GTR + F + R4
*nad4*_pos1	TVM + F + R8	*nad4*_pos3	TIM3 + F + I + I + R6
*nad4*_pos2	GTR + F + R7	*nad4L*_pos2	TIM + F + I + I + R3
*nad4*_pos3	GTR + F + ASC + R9	*nad4L*_pos3	TPM2u + F + R4
*nad4L*_pos1	GTR + F + I + I + R6	*nad1*_pos2	TVM + F + I + I + R4
*nad4L*_pos2	GTR + F + I + I + R5	*nad1*_pos3	TPM2u + F + R5
*nad6*_pos1	TIM2 + F + R9	*rrnL*	GTR + F + I + I + R4
*nad6*_pos2	TVM + F + I + I + R7	*rrnS*	GTR + F + R4
*nad6*_pos3	GTR + F + ASC + R9		
*cob*_pos1	GTR + F + I + I + R8		
*cob*_pos2	TVM + F + R6		
*cob*_pos3	TIM2 + F + ASC + R11		
*nad1*_pos1	GTR + F + R7		
*nad1*_pos3	TIM + F + ASC + R10		
*rrnL*	GTR + F + I + I + R7		
*rrnS*	GTR + F + R7		
*(c) Dataset 3 (13PCGs_AA)*			
*nad2_atp6_nad*3	mtMet + F + I + I + R10	*nad2_cox1_cox2_nad4L_cob_nad1*	mtART + F + I + I + R5
*cox1_cox3_cob*	mtInv + R9	*atp8_atp6_cox3_nad3_nad5*	mtART + F + R5
*cox2*	mtMet + I + I + R8	*nad4_nad6*	mtART + F + I + I + R5
*atp8*	mtMet + F + I + I + R7		
*nad5*_nad4_nad4L_nad1	mtMet + F + I + I + R10		
*nad6*	mtMet + F + R10		

### Supplementary Information

**Additional file 1: Table S1.** List of mitochondrial genomes of Lepidoptera (including *A. conjugella* mitochondrial genome) investigated in the study, representing 18 superfamilies and 42 families (Supplementary Figure S2), including outgroups species ( *Phryganea cinerea*, *Phryganopsyche latipennis*, *Cheumatopsyche brevilineata*, *Limnephilus hyalinus*, and *Stenopsyche angustata*).

**Additional file 2: Table S2.** Results of two-tailed tests of substitution saturation for each codon position of the 13 PCGs. The following abbreviations were used: index of substitution saturation (ISS), critical value of ISS supposing symmetrical cladogenesis (ISS.CSym.), critical value of ISS supposing asymmetrical cladogenesis (ISS.CAsym.).

**Additional file 3: Figure S1.** Maximum Likelihood phylogenetic tree based on 13 PCGs of 56 mitogenomes including outgroups species (*Phryganea cinerea*, *Phryganopsyche latipennis*, *Cheumatopsyche* *brevilineata*,* Limnephilus hyalinus*, and *Stenopsyche angustata*).

**Additional file 4: Figure S2.** Maximum Likelihood phylogenetic tree based on 13 PCGs + 2 rRNAs compared *A. conjugella* mitochondrial genome with the mitochondrial genomes of 507 Lepidoptera obtained from GenBank, representing 18 superfamilies and 42 families (Supplementary Table S1), including outgroups species (*Phryganea cinerea*, *Phryganopsyche latipennis*, *Cheumatopsyche brevilineata*, *Limnephilus hyalinus*, and *Stenopsyche angustata*).

## Data Availability

*A. conjugella*. mitochondrial genome has been deposited in GenBank under accession: ON496993; Fig. 1 (https://github.com/Simo-N-Maduna/Mito-Phylogenomics/tree/main/Mitophylogenomics_PartII_Lepidoptera). The 507 mitogenomes from the study were downloaded from GenBank. Their accession numbers and references are listed in Table S1. Other supporting results are included within the article and its additional files.
