# Supplementary figures and images for: Novel insight into lepidopteran phylogenetics from the mitochondrial genome of the apple fruit moth of the family Argyresthiidae
Source: BMC Genomics. 2024 Jan 2;25:21. doi: 10.1186/s12864-023-09905-1 (PMC10759517; doi:10.1186/s12864-023-09905-1)

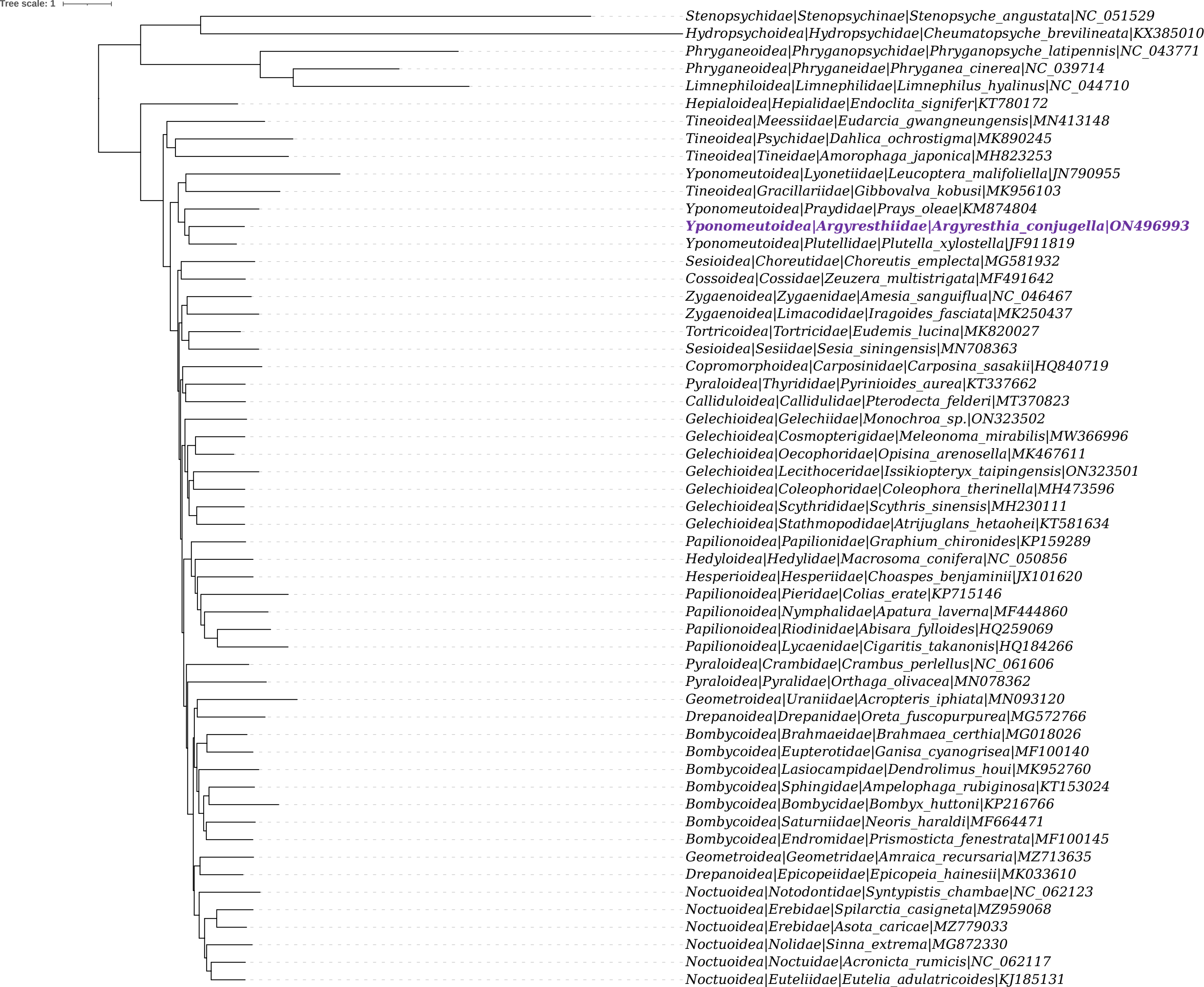

Supplement: Supplementary file 3 — Additional file 3: Figure S1. Maximum Likelihood phylogenetic tree based on 13 PCGs of 56 mitogenomes including outgroups species (Phryganea cinerea, Phryganopsyche latipennis, Cheumatopsyche brevilineata, Limnephilus hyalinus, and Stenopsyche angustata). [file 12864_2023_9905_MOESM3_ESM.pdf]
