# Supplementary material for: Novel insight into lepidopteran phylogenetics from the mitochondrial genome of the apple fruit moth of the family Argyresthiidae
Source: BMC Genomics. 2024 Jan 2;25:21. doi: 10.1186/s12864-023-09905-1 (PMC10759517; doi:10.1186/s12864-023-09905-1)

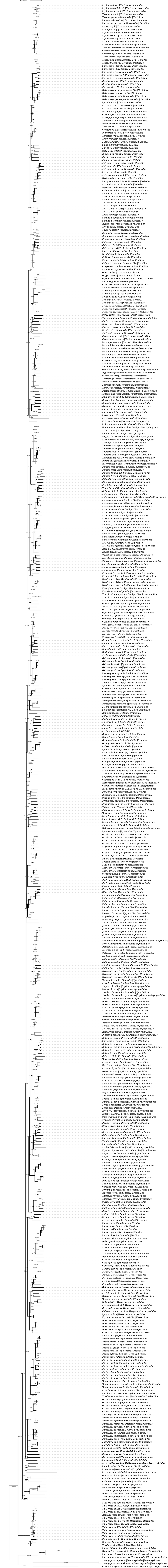

Supplement: Supplementary file 4 — Additional file 4: Figure S2. Maximum Likelihood phylogenetic tree based on 13 PCGs + 2 rRNAs compared A. conjugella mitochondrial genome with the mitochondrial genomes of 507 Lepidoptera obtained from GenBank, representing 18 superfamilies and 42 families (Supplementary Table S1), including outgroups species (Phryganea cinerea, Phryganopsyche latipennis, Cheumatopsyche brevilineata, Limnephilus hyalinus, and Stenopsyche angustata). [file 12864_2023_9905_MOESM4_ESM.pdf]
